# Effects of site elevation and grazing exclusion on phenolic compound production in *Nardus stricta* plants in high-elevation grasslands

**DOI:** 10.1371/journal.pone.0330638

**Published:** 2025-09-10

**Authors:** Jorge Durán, Xoaquín Moreira, Marta Correia, Ana Cao, Joana Serôdio, Susana Rodríguez-Echeverría, Alexandra Rodríguez

**Affiliations:** 1 Misión Biológica de Galicia (MBG-CSIC), Pontevedra, Galicia, Spain; 2 Department of Life Sciences, Centre for Functional Ecology, Associate Laboratory TERRA, University of Coimbra, Coimbra, Portugal,; 3 Instituto Mediterráneo de Estudios Avanzados (IMEDEA-CSIC-UIB), Esporles, Islas Baleares, España, Spain; University of Udine: Universita degli Studi di Udine, ITALY

## Abstract

Specialized plant metabolism, particularly phenolic compound production, contributes significantly to the functioning and resilience of mountain ecosystems. Livestock grazing can influence phenolic production, with its effects varying depending on microclimatic factors and soil conditions. Despite the ecological significance of this process, the impact of livestock grazing on phenolic production in alpine plants remains insufficiently explored. To address this knowledge gap, we conducted a field experiment to investigate the individual and combined effects of site elevation and experimental grazing exclusion on phenolic compound production in *Nardus stricta* plants. After two growing seasons, we collected leaf samples to quantify phenolic diversity and concentration. Site elevation significantly affected phenolic richness and flavonoid concentrations, with higher levels at the highest elevation compared to lower sites. However, livestock grazing exclusion had no significant impact on phenolic concentration, diversity, or any interactions between grazing exclusion and site elevation across sites. This study enhances understanding of plant chemistry and responses to stressors, offering insights into plant adaptations to environmental and land-use changes.

## Introduction

Mountain ecosystems provide essential ecosystem services such as carbon storage, water regulation, and biodiversity conservation [1,2]. They also serve as grazing grounds critical for local livestock and the production of high-quality dairy and meat products [3]. However, these ecosystems are particularly fragile due to environmental factors like low temperatures and nutrient-poor soils, making them highly sensitive to various disturbances [4]. Rising temperatures and increased drought conditions, for example, are significantly affecting plant phenology, distribution, and productivity [5]. Overgrazing exacerbates soil degradation, reduces plant biomass, and alters community composition, undermining ecosystem stability [6]. Conversely, grazing abandonment leads to shrub encroachment, biodiversity loss, and disruptions to nutrient cycles [7]. Together, these pressures threaten the health and resilience of fragile mountain environments, underscoring the need for effective management to preserve their vital ecological functions.

Plant specialized metabolism, particularly the production of phenolic compounds, contributes significantly to plant fitness and the functioning and resilience of mountain ecosystems [8]. These phenolic compounds help protect plants against herbivores, pathogens, and environmental stressors such as ultraviolet radiation and extreme temperature fluctuations at high elevations [9,10], thus enhancing ecosystem resilience. For instance, the diversity and concentration of phenolic compounds—especially flavonoids and tannins—have been observed to increase with elevation in several species, including oaks (*Quercus* spp.) and *Vicia* plants, likely as an adaptive response to increased UV exposure and harsher climatic conditions [11,12]. Furthermore, phenolic compounds have significant implications for the health and product quality of livestock grazing in mountain ecosystems [13,14]. These compounds contribute to the antioxidant content of milk and meat, enhancing their nutritional value and potentially offering health benefits to consumers [15]. However, excessive phenolic concentrations can negatively affect livestock fertility and metabolism, creating a complex balance between ecological and economic considerations [16]. While livestock grazing has a notable impact on the production and diversity of phenolic compounds, these changes are likely being highly context-dependent [17–19]. Factors such as microclimatic conditions—like temperature, moisture, and solar radiation [20,21]—as well as soil attributes, like nutrient availability and pH [22,23], influence phenolic content. In high mountain environments, where extreme conditions amplify these effects, grazing might have more pronounced impacts on plant metabolism and the accumulation of phenolic compounds. Despite its ecological importance, the role of livestock grazing in modulating phenolic production in alpine and sub-alpine plants remains an underexplored area of research, warranting further investigation to better understand the interplay between grazing, plant metabolism, and ecosystem dynamics.

To address this knowledge gap, we conducted a field experiment in sub-alpine grasslands of Serra da Estrela (Portugal) to examine the individual and combined effects of site elevation and experimental grazing exclusion on phenolic compound production in *Nardus stricta* L. plants. After two growing seasons, we collected leaf samples to quantify phenolic compound concentrations. Our primary research questions were: (i) Do the diversity and abundance of phenolic compounds in leaves vary across sites with different elevations? (ii) Do the diversity and abundance of phenolic compounds respond to grazing exclusion? (iii) Do the effects of grazing exclusion vary across elevational sites, indicating potential interactions or dependencies across scales? This study will deepen our understanding of plant chemistry and their responses to both environmental and anthropogenic stressors, providing valuable insights that can inform predictions of plant adaptations to changing environmental conditions and land-use practices.

## Materials and methods

### Experimental design and sampling

**Fig 1:**
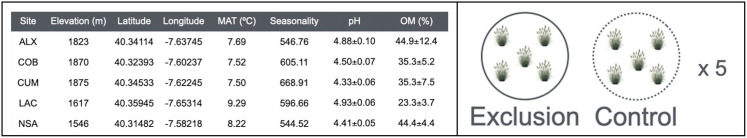
The five high-elevation grasslands in Serra da Estrela, Portugal, where our study was conducted: Malhõesitos da Talada (ALX), Covão do Boi (COB), Cume (CUM), Lagoa Comprida (LAC), and Nave Santo António (NSA). For each site, we provide detailed information about elevation, latitude, longitude, climate data such as the mean annual temperature (MAT) and temperature seasonality (calculated as the standard deviation × 100), and soil conditions such as pH and organic matter (OM) content. For the experimental design, we established five plots at each site (10 × 10 meters) with livestock exclusion structures and control areas, resulting in 50 experimental units (5 sites × 2 grazing treatments × 5 plots).

The study was conducted in five high-elevation *Nardus stricta* grasslands within Serra da Estrela, Portugal (Fig 1), a habitat designated as a priority under the European Union’s Natura 2000 directive (Annex I, code 6230). The studied grasslands are located at elevations ranging from 1545 to 1875 m a.s.l. in the Serra da Estrela mountain range (Fig 1), which represents one of the highest points in the Iberian Peninsula. Although 1875 m would be considered mid- or even low elevation in a global context (e.g., the Himalayas), it is relatively high regionally; thus, for the purposes of this study, we refer to 1875 m as a high-elevation site, reflecting the local ecological and climatic conditions at this altitude. These grasslands exhibit moderate variation in climatic and soil characteristics (see below and Fig 1). Their soils are classified as acidic umbrisols, which support the dominance of *N. stricta*, a grass species that makes up approximately 80–90% of the plant community. The study sites are characterized by free-ranging cattle, goats, and mainly sheep that graze on *Nardus stricta*, providing a natural setting to examine grazing effects on plant chemical defenses across contrasting environmental conditions. This species, which dominates the plant community, is considered poorly palatable but is nevertheless the most consumed forage resource in the area. This apparent contradiction reflects that its high abundance compensates for its low relative palatability, making it a primary target of herbivory. Consequently, grazing pressure on *N. stricta* is ecologically relevant, as it can strongly influence plant community structure and ecosystem dynamics.

In October 2020, we selected five plots at each site for our experiment. Each plot was located on flat terrain (no slope) and measured 10 × 10 meters (Fig 1). Within each plot, we installed a livestock exclusion structure (LES) to examine grazing impacts on high-altitude grasslands (Fig 1). Each LES measured 1.5 m in height with a diameter of 0.90 m and contained several *N. stricta* individuals. In addition to the LES, we established a control area of the same size nearby in each plot, which also contained several *N. stricta* individuals (Fig 1). This resulted in 50 experimental units (5 sites × 2 grazing treatments × 5 plots) (Fig 1).

In July 2022, two years after grazing exclusion and during the peak of the growing season, five *N. stricta* tussocks were randomly selected from each experimental unit, totalling 250 tussocks (i.e., 50 experimental units × 5 tussocks). From each tussock, five fully expanded leaves with minimal or no insect herbivore damage were collected, ensuring minimal variation due to metabolite induction. Leaf age was standardized based on position along the branch, colour, and texture. The leaves were transported in paper bags to the laboratory and oven-dried at 40°C for 48 hours to prepare for chemical analysis of phenolic compounds.

Fieldwork and sample collection were conducted under permits granted by the Instituto da Conservação da Natureza e das Florestas (ICNF), the national authority responsible for managing protected areas and natural resources in Portugal.

### Quantification of phenolic compounds

For each plant, we employed a ball mill to grind the five collected leaves and extracted phenolic compounds from 20 mg of dry, pulverized leaf tissue using 1 mL of 70% methanol in an ultrasonic bath for 15 minutes, followed by centrifugation [24]. The resulting extracts were then transferred to chromatographic vials. To quantify the phenolic compounds, we used an ultra-high-performance liquid chromatography system (UHPLC, Nexera LC-30AD; Shimadzu), equipped with an M20A UV/VIS photodiode array detector (PDA; SPD-M20A, Shimadzu). Compound separation was achieved on a Kinetex™ Core-Shell C18 column (2.6 µm, 100 × 4.6 mm; Phenomenex), protected with a C18 guard cartridge. The flow rate was set to 0.4 mL min^−1^, and the column oven temperature was maintained at 25 ºC. The mobile phase consisted of two solvents: water + formic acid (0.1%) (A) and acetonitrile (B). The gradient started with 7% B, increased to 30% B at 30 minutes, 50% B at 45 minutes, 60% B at 46 minutes, 100% B at 50 minutes, and returned to 7% B at 53 minutes. Phenolic compounds were detected at 330 nm.

For phenolic compound identification, we used a UHPLC-PDA system (Thermo Dionex Ultimate 3000), coupled with an electrospray ionization quadrupole time-of-flight mass spectrometer (QTOF-MS/MS, Bruker Compact™), using the same chromatographic conditions as for quantification. Mass spectra (MS) and MS^2^ were acquired in full scan mode, with negative ionization. Only two groups of phenolic compounds were detected in our samples: (i) flavonoids (e.g., luteolin, tricin derivatives) and (ii) hydroxycinnamic acids (e.g., ferulic, coumaric, and caffeic acids derivatives) (see S1 Table in the Supplementary Material). Identification was based on the comparison of parent ion mass, fragmentation pattern, and UV spectra with literature data and public databases such as PubChem (https://pubchem.ncbi.nlm.nih.gov). Flavonoids were quantified as rutin equivalents, and hydroxycinnamic acids as ferulic acid equivalents using external calibration curves. See Moreira et al. [25] for a detailed explanation of compound separation, identification, and quantification. We also quantified phenolic compound diversity at the individual plant level using two indices: the total number of phenolic compounds, or phenolic richness (S) and the Shannon–Weiner index (H). The latter index was calculated as H = – Σ(P_i_ log[P_i_]), where P_i_ is the relative amount of a given phenolic compound divided by the total phenolics in each plant. Both indices play a key role in boosting a plant’s ability to resist biotic threats such as herbivores and pathogens [26,27]. Specifically, a greater diversity of phenolic compounds allows plants to produce a wider range of chemical defences, offering enhanced protection against herbivory [26].

### Statistical analyses

First, we ran general linear mixed models to test the effects of site (five levels: ALX, COB, CUM, LAC, NSA), grazing treatment (two levels: control vs. grazing exclusion), and their interaction on the diversity (S and H) and abundance of phenolic compounds. We initially analyzed each phenolic compound separately and applied a Bonferroni correction to control for Type I error inflation. After this adjustment, grazing had no significant effect on the concentration of any individual compound. Given the lack of significant responses and the inherent complexity of interpreting patterns at the individual compound level, we chosed to present and discuss our findings using richness and diversity metrics. These metrics not only provide an integrative view of phenolic composition but also implicitly capture variation at the compound level. Abundance was analysed separately for flavonoids and hydroxycinnamic acids. The interaction between site and grazing treatment assessed whether the effects of grazing exclusion varied across sites at different elevations, revealing a potential cross-scale dependency in plant responses to environmental and grazing pressures. To account for site and grazing effects with the correct error structure, we included a three-way random effect for plot, site, and grazing treatment in all models.

All analyses were conducted using PROC MIXED in SAS version 9.4 (SAS Institute, Cary, NC) [28]. To achieve normality of the residuals, we log-transformed the data for flavonoids and hydroxycinnamic acids and reported model least-squares means and standard errors as descriptive statistics.

## Results and discussion

**Fig 2:**
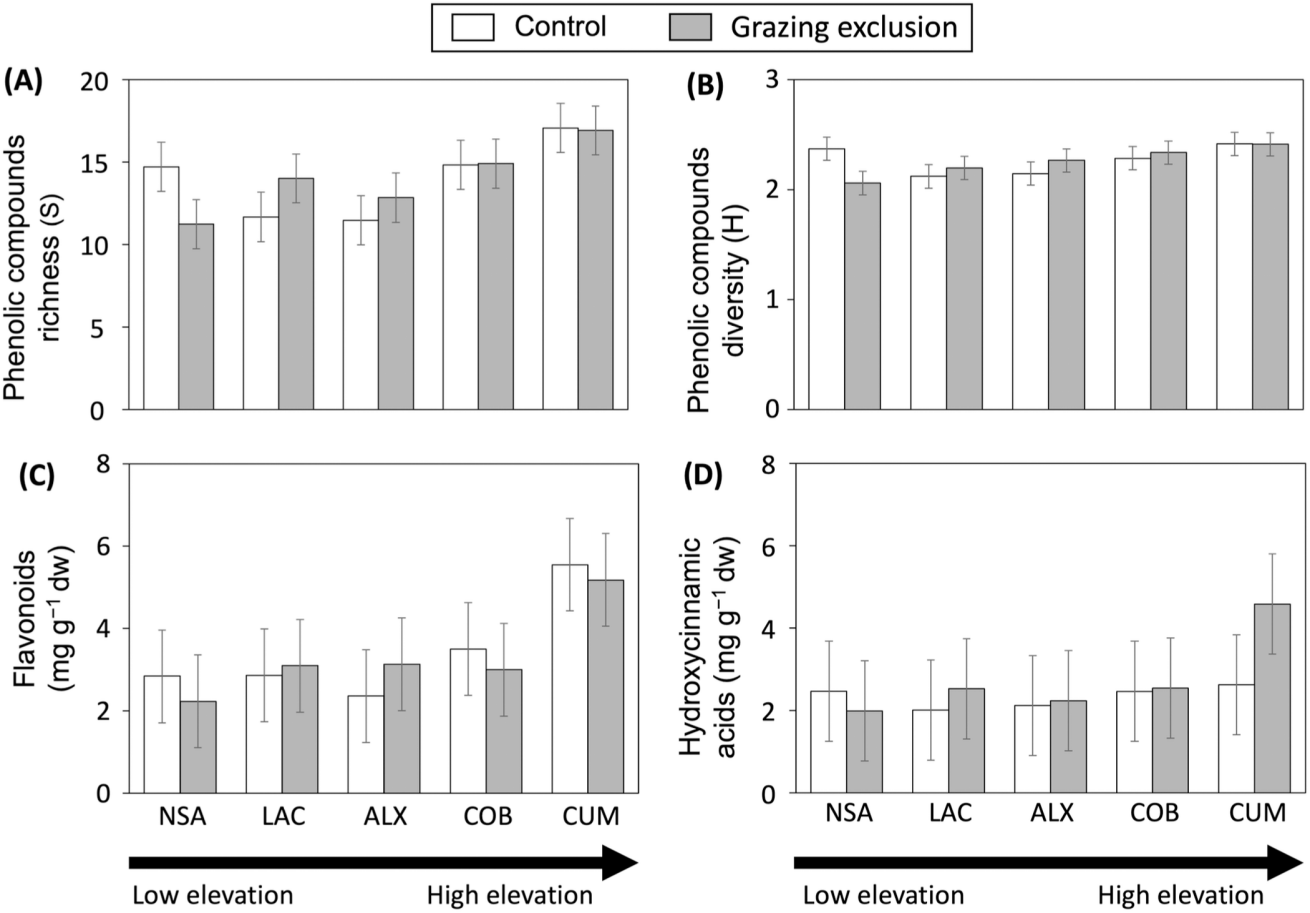
The effects of grazing exclusion on phenolic compound production in *Nardus stricta* leaves across five sites with varying elevations in central Portugal are shown. Panel (A) illustrates the total number of phenolic compounds, or phenolic richness (S), while panel (B) presents the Shannon–Weiner index (H), a measure of phenolic diversity. The concentrations (in mg g⁻¹ d.w.) of flavonoids and hydroxycinnamic acids are shown in panels (C) and (D), respectively. Plants subjected to grazing exclusion are represented by gray bars, while control plants are indicated by white bars. The bars represent least square means ± standard errors for phenolic richness and diversity (panels A and B), and back-transformed least square means ± standard errors for flavonoid and hydroxycinnamic acid concentrations (panels C and D), derived from linear models. Statistical results are summarized in Table 1.

The site significantly affected phenolic richness (S) and the concentration of flavonoids but did not influence diversity (H) or the concentration of hydroxycinnamic acids (Table 1, Fig 2A–D, S1 Fig). In particular, plants growing at the highest elevation site (CUM) exhibited greater concentrations and richness of phenolic compounds in their leaves compared to those growing at the lowest elevation sites (LAC and NSA). High elevations are linked to increased exposure to solar radiation and more extreme temperature fluctuations [4]. These stressors often trigger enhanced synthesis of phenolic compounds, which act as protective antioxidants against ultraviolet-induced oxidative damage [e.g., 20,29]. Additionally, lower atmospheric pressure and reduced oxygen levels at higher elevations may further stimulate the production of secondary metabolites, including phenolics, as part of the plant’s adaptive mechanisms to harsh environmental conditions [30]. Notably, our results reveal differential responses of phenolic compounds to environmental conditions. Flavonoids, which are often produced in response to stressors such as UV radiation or nutrient availability, may increase under site-specific pressures [31], while hydroxycinnamic acids show less variability, possibly due to their structural roles or more tightly controlled biosynthesis [32]. However, these patterns are not universal. Some studies have reported that phenolic production does not increase under stress and may even decline, depending on species, developmental stage, or local resource availability [33]. This suggests that additional factors—such as soil nutrient status, plant ontogeny, or genotype-by-environment interactions—may modulate the synthesis and accumulation of phenolic compounds in response to environmental stress. Additionally, the lack of significant changes in diversity (H) suggests that, despite the production of new compounds, their relative proportions remain stable, maintaining overall chemical composition and complexity. Overall, these findings highlight how elevation-related environmental stressors selectively shape the richness and concentration of specific phenolic compounds, particularly flavonoids, while leaving overall chemical diversity and structurally essential compounds like hydroxycinnamic acids relatively unchanged.

**Table 1:** Effects of site, grazing treatment (two levels: control vs. grazing exclusion), and their interaction on the total number of phenolic compounds (phenolic richness, S), the Shannon–Weiner index (H), and the concentrations of flavonoids and hydroxycinnamic acids in leaves of *Nardus stricta* plants located at five sites varying in elevation. In all models, we included the three-way interaction between plot, site, and grazing treatment as a random effect to appropriately account for error terms while analysing the main factors (i.e., site and grazing treatment). To achieve normality of the residuals, we log-transformed the data for flavonoids and hydroxycinnamic acids. F-values with degrees of freedom (numerator, denominator) and associated significance levels (*P-value*s) are provided. Significant *P*-values (*P* < 0.05) are indicated in bold.

	Site (S)		Grazing (G)		S × G	
	F_4,40_	*P*	F_1,40_	*P*	F_4,40_	*P*
Phenolic richness (S)	3.49	**0.015**	0.00	0.976	1.10	0.371
Shannon diversity index (H)	1.83	0.142	0.05	0.832	1.31	0.284
Flavonoids	12.66	**<0.001**	0.07	0.788	1.52	0.216
Hydroxycinnamic acids	1.99	0.115	1.13	0.295	1.08	0.380

Our results indicated no significant effects of livestock grazing exclusion on the phenolic concentration or diversity in leaves of *N. stricta* plants (Table 1, Fig 2A–D, S1 Fig). In addition, we did not observe a site-by-grazing interaction for flavonoid and hydroxycinnamic acid concentrations, nor any effect on phenolic diversity (S, H) (Table 1, Fig 2A–D, S1 Fig), indicating that the non-significant impact of grazing exclusion was consistent across the different sites. This lack of response to grazing exclusion may be explained by several factors. First, *N. stricta* may have developed compensatory mechanisms that allow it to maintain phenolic concentrations despite grazing pressure [34,35], including shifts in other plant traits. Indeed, *N. stricta* is known to be poorly palatable, with a primary defence against herbivory being the accumulation of silica in its leaves [36–38]. This mechanism, along with others such as increased production of terpenoids or adjustments in nutrient allocation, may help mitigate the effects of grazing exclusion [39]. Second, the intensity of grazing pressure at our study sites may not have been high enough to induce significant changes in phenolic production. If grazing pressure was mild, the exclusion of livestock might not have notably altered plant defences or metabolism [40]. Finally, phenolic concentrations may take longer to respond to grazing changes [41]. The two-year duration of grazing exclusion may not have been long enough to observe substantial shifts in phenolic profiles [42,43]. For example, Turley, Odell (42) found that *Rumex acetosa* L. (Polygonaceae) plants showed differences in secondary metabolite concentrations (tannins and oxalate) after 26 years of rabbit exclusion.

Future research should focus on examining the long-term effects of environmental stressors, such as solar radiation and temperature fluctuations, on phenolic compound production across various elevations and plant systems. Investigating how these abiotic factors influence plant chemical defences will deepen our understanding of how plants adapt to environmental variation, especially at high elevations where stressors are more pronounced. Additionally, future studies should explore the role of grazing pressure in shaping plant secondary metabolism over longer periods. Mountain ecosystems provide essential ecosystem services, but their fragility and sensitivity to disturbances, such as climate change, overgrazing, and grazing abandonment, can threaten their ecological stability and require effective management to ensure their resilience. Our study suggests that grazing exclusion do not have an immediate impact on phenolic concentrations or diversity, at least in our study site, possibly due to mild grazing pressure or specific compensatory mechanisms in *N. stricta*. However, secondary metabolite responses to grazing may require more time to manifest, as shown in previous studies where long-term exclusion led to measurable shifts in plant chemistry. Therefore, extending the duration of grazing exclusion experiments and considering varying intensities of grazing would provide valuable insights into how grazing affects plant defence strategies over time. Moreover, examining a broader range of plant species across different ecological contexts could help determine whether grazing exclusion effects are species-specific or influenced by site characteristics, further enhancing our understanding of plant-herbivore interactions and plant responses to environmental change. Finally, it is important to note that the elevational gradient examined in this study spans less than 500 meters, which is relatively modest compared to many other elevational studies that typically investigate changes across gradients of 1000 meters or more [e.g., 12,44]. Future research should aim to include steeper elevational gradients to better capture broader environmental variation and more pronounced ecological responses.

## Supporting information

S1 Table Detailed information on the MS spectra of the identified compounds. (PDF)

S1 Fig The effects of grazing exclusion on phenolic compound production in Nardus stricta leaves across five sites with varying elevations in central Portugal are shown. Panel (A) illustrates the total number of phenolic compounds, or phenolic richness (S), while panel (B) presents the Shannon–Weiner index (H), a measure of phenolic diversity. The concentrations (in mg g⁻¹ d.w.) of flavonoids and hydroxycinnamic acids are shown in panels (C) and (D), respectively. Plants subjected to grazing exclusion are represented by blue bars, while control plants are indicated by red bars. The bars represent mean values ± standard errors.
(TIFF)
