# Supplementary material for: Effects of site elevation and grazing exclusion on phenolic compound production in Nardus stricta plants in high-elevation grasslands
Source: PLoS One. 2025 Sep 10;20(9):e0330638. doi: 10.1371/journal.pone.0330638 (PMC12422442; doi:10.1371/journal.pone.0330638)
Supplement: S1 Table — (PDF) [file pone.0330638.s001.pdf]

RT (min)	λmax	[M-H]- (m/z)	MS/MS [M-H]- (m/z)	Compound	Phenolic group	Reference
7.28	290, 324	353.0888	191.056, 179.034, 155.031, 135.045, 127.039	caffeoyl isocitric acid or caffeoyl quinic acid	hydroxycinnamic acid	https://www.ncbi.nlm.nih.gov/pmc/articles/PMC5380550/
10.22	290, 325	.	.	caffeoyl isocitric acid or caffeoyl quinic acid	hydroxycinnamic acid	
10.52	290, 325	353.0889	191.056, 179.034, 173.044, 161.023, 155.032, 135.045, 127.039, 111.044, 93.034	caffeoyl isocitric acid or caffeoyl quinic acid	hydroxycinnamic acid	https://www.ncbi.nlm.nih.gov/pmc/articles/PMC5380550/
10.75	290, 325	353.0889	191.056, 179.034, 173.044, 161.023, 155.032, 135.045, 127.039, 111.044, 93.034	caffeoyl isocitric acid or caffeoyl quinic acid	hydroxycinnamic acid	https://www.ncbi.nlm.nih.gov/pmc/articles/PMC5380550/
12.68	287, 316	419.1069	353.09, 257.08, 191.05, 179.03, 161.02	caffeoyl quinic acid derivative	hydroxycinnamic acid	https://www.ncbi.nlm.nih.gov/pmc/articles/PMC5380550/
13.5	270 345	579.1359	519.115, 489.101, 459.090, 429.082, 399.072, 369.061	Luteolin-6-C-arabinoside-8-C- glucoside	flavonoid	https://www.ncbi.nlm.nih.gov/pmc/articles/PMC9943761/
14.42	269, 342	579.135	519.115, 489.101, 459.090, 429.082, 399.072, 369.061	Luteolin-6-C-arabinoside-8-C- glucoside	flavonoid	https://www.ncbi.nlm.nih.gov/pmc/articles/PMC9943761/
14.61	273, 328	367.1004	193.04, 173.04, 155.03, 134.03, 111.04, 93.03	feruloyl isocitric acid	hydroxycinnamic acid	https://www.ncbi.nlm.nih.gov/pmc/articles/PMC5380550/
15.28	327	443.1165	267.07, 249.06, 193.05, 175.03, 134.03, 113.02, 85.02	Diferuloyl glycerol	hydroxycinnamic acid	https://doi.org/10.1016/j.foodchem.2016.05.052
15.07	271, 341	563.1398	545.13, 603.12, 473.11, 443.09, 383.07, 353.06	apigenin xyloside glucoside	flavonoid	10.3390/ijms151120668
15.7	267, 346	609.1481	519.11, 489.12, 429.12, 357.09, 327.08, 309.07, 285.04	luteolin-6-C-glucoside-7-O-glucoside	flavonoid	https://doi.org/10.1016/j.bjp.2016.10.003
15.82	289, 319	925.2331	839.23, 719.18, 677.17, 559.15, 515.14, 395.10, 335.07, 179.03	caffeoyl quinic acid derivative	hydroxycinnamic acid	
15.86	269, 346	447.0924	429.081, 357.060, 327.049, 297.039, 285.0399	luteolin hexoside	flavonoid	https://pubchem.ncbi.nlm.nih.gov/compound/Lutonarin#section=LC-MS
16.16	284, 326	677.1831	515.19, 353.12, 341.12, 179.05	tricaffeoylquinic acid	hydroxycinnamic acid	
	311	237.077	163.037, 145.028, 119.048, 117.033	coumaroyl glycerol	hydroxycinnamic acid	https://doi.org/10.1016/j.foodchem.2016.05.052
17.49	397, 322	677.2398	515.18, 353.12, 341.12, 335.10, 191.07, 179.05,173.06, 161.03	tricaffeoylquinic acid	hydroxycinnamic acid	
18.81	269, 331	581.2449	145.028	sinapic acid hexoside derivative	hydroxycinnamic acid	
	296, 323	763.1748	720.24,677.23, 557.19, 515.17, 395.13, 353.11, 233.09, 179.05	tricaffeoylquinic acid derivative	hydroxycinnamic acid	
18.74	269, 349	681.1953	351.057, 329.066, 315.051, 307.06, 289.0584, 193.03, 175.024, 157.02, 113.02	glucuronopyranoside	flavonoid	doi:10.3390/molecules21091229
20.02	253, 366, 346	447.0945	327.06, 285.06	Luteolin hexoside	flavonoid	doi: 10.3390/plants12051001.
20.7	298, 323	763.2514	601.24, 557.20, 515.18, 395.13, 353.12, 341.12, 335.10, 233.08, 215.07, 179.05, 173.06, 161.04	malonyl tricaffeoylquinic acid	hydroxycinnamic acid	https://doi.org/10.1002/rcm.4585
21.13	267, 343	491.1205	371.056, 329.066, 314.043	tricin glucoside	flavonoid	https://doi.org/10.1039/C5GC03062E
21.56	292.326	515.1211	353.12, 335.11, 191.07, 179.05, 173.06, 161.03	dicaffeoyl quinic acid	hydroxycinnamic acid	
23.03	292. 326	515.1219	353.12, 335.11, 191.07, 191.07, 179.05, 173.06, 161.03, 155.05, 135.05	dicaffeoyl quinic acid	hydroxycinnamic acid	
23.02	269.341	887.194	663.119, 557.112, 399.090, 351.054, 333.0455, 329.065, 315.0484, 289.059, 271.042, 223.059, 175.023, 133.013, 113.023, 72.992	Tricin -O-sinapoyl-glucuronopyranosyl-O-glucuronopyranoside	flavonoid	doi:10.3390/molecules21091229
23.45	272, 332	873.2143	649.138, 573.123, 543.134, 491.115, 329.065, 223.059, 205.048, 157.014	Tricin sinapoyl derivative	flavonoid	doi:10.3390/molecules21091229
23.72	270, 337	491.119	476.093, 461.071, 343.045, 329.063, 328.057, 313.035	Tricin hexoside	flavonoid	https://doi.org/10.1039/C5GC03062E
24.37	270, 336	857.1815	663.11, 527.10, 369.08, 351.05, 333.045, 329.06, 315.04, 289.06, 193.05, 175.02, 157.02	glucuronopyranoside	flavonoid	doi:10.3390/molecules21091229
24.72	270, 330	687.1944	491.11, 329.06, 195.06, 165.05	Tricin hexoside derivative	flavonoid	https://doi.org/10.1039/C5GC03062E
25.47	290, 326	601.1227	515.16, 395.13.353.12, 335.10, 233.09, 191.07, 179.05, 173.06, 161.03	malonyl dicaffeoylquinic acid	hydroxycinnamic acid	https://doi.org/10.1002/rcm.4585
26.28	290, 328	601.1207	395.13, 353.12, 233.08, 191.07, 179.05, 173.06, 161.03	malonyl dicaffeoylquinic acid	hydroxycinnamic acid	https://doi.org/10.1002/rcm.4585
36.49	270, 336	329.0638	314.04, 299.02, 371.02	Tricin	flavonoid	https://doi.org/10.1039/C5GC03062E
36.79	271, 332	525.1396	477.11, 239.06, 314.04, 195.06, 165.05	Guaiacylglyceryl tricin	flavonoid	https://doi.org/10.1039/C5GC03062E
37.01	290, 323	819.2343	193.04, 175.04, 160.01	di-O-feruloyl-tri-O-acetyl sucrose	hydroxycinnamic acid	https://nph.onlinelibrary.wiley.com/doi/full/10.1111/nph.15520
37.98	270, 331	525.1405	477.11, 239.06, 314.04, 195.06, 165.05	Guaiacylglyceryl tricin	flavonoid	https://doi.org/10.1039/C5GC03062E
38.15	295, 315	413.1219	193.05, 175.03, 163.03, 145.02, 134.03, 119.05	feruloyl coumaroyl glycerol	hydroxycinnamic acid	https://doi.org/10.1016/j.foodchem.2016.05.052
38.49	295, 323	443.1759	428.11, 267.08, 249.07, 207.06, 193.05, 175.03, 160.01, 149.06, 134.03, 117.03	diferuloyl glycerol	hydroxycinnamic acid	https://doi.org/10.1016/j.foodchem.2016.05.052
