# Supplementary figures and images for: Effects of site elevation and grazing exclusion on phenolic compound production in Nardus stricta plants in high-elevation grasslands
Source: PLoS One. 2025 Sep 10;20(9):e0330638. doi: 10.1371/journal.pone.0330638 (PMC12422442; doi:10.1371/journal.pone.0330638)

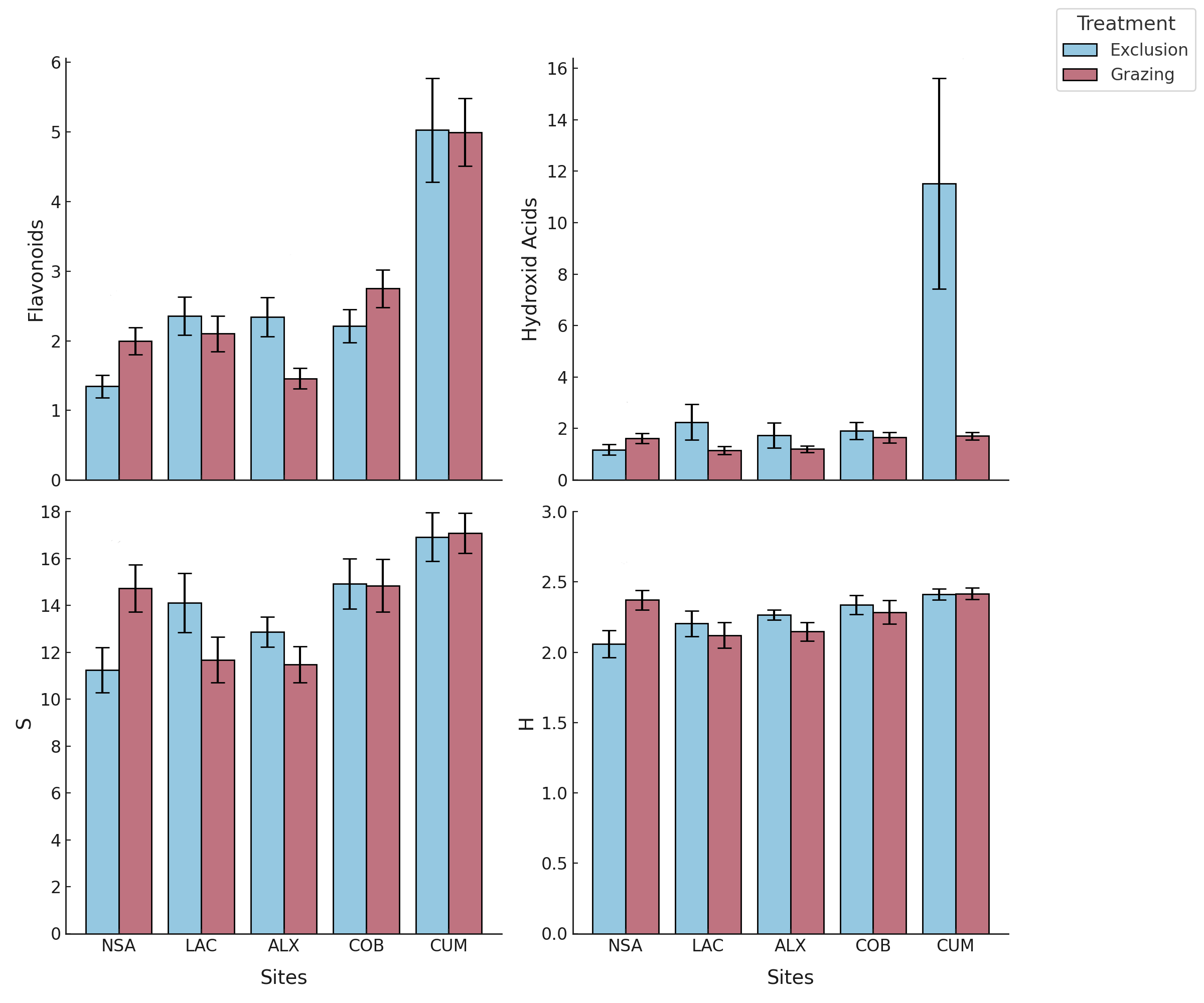

Supplement: S1 Fig — Panel (A) illustrates the total number of phenolic compounds, or phenolic richness (S), while panel (B) presents the Shannon–Weiner index (H), a measure of phenolic diversity. The concentrations (in mg g⁻¹ d.w.) of flavonoids and hydroxycinnamic acids are shown in panels (C) and (D), respectively. Plants subjected to grazing exclusion are represented by blue bars, while control plants are indicated by red bars. The bars represent mean values ± standard errors. (TIFF) [file pone.0330638.s002.tiff]
